# Gene co-expression architecture in peripheral blood in a cohort of remitted first-episode schizophrenia patients

**DOI:** 10.1038/s41537-022-00215-1

**Published:** 2022-04-27

**Authors:** Natalia Rodríguez, Patricia Gassó, Albert Martínez-Pinteño, Àlex-González Segura, Gisela Mezquida, Lucia Moreno-Izco, Javier González-Peñas, Iñaki Zorrilla, Marta Martin, Roberto Rodriguez-Jimenez, Iluminada Corripio, Salvador Sarró, Angela Ibáñez, Anna Butjosa, Fernando Contreras, Miquel Bioque, Manuel-Jesús Cuesta, Mara Parellada, Ana González-Pinto, Esther Berrocoso, Miquel Bernardo, Sergi Mas, 2EPS group, Silvia Amoretti S, Constanza Moren, Carol Stella, Xaquin Gurriarán, Anna Alonso-Solís, Eva Grasa, Jessica Fernandez, Itxaso Gonzalez-Ortega, Francesc Casanovas, Antoni Bulbuena, Ágatha Núñez-Doyle, Olga Jiménez-Rodríguez, Edith Pomarol-Clotet, Isabel Feria-Raposo, Judith Usall, Daniel Muñoz-Samons, Jose L. Ilundain, Ana Maria Sánchez-Torres, Jeronimo Saiz-Ruiz, Isabel López-Torres, Juan Nacher, Concepción De-la-Cámara, Miguel Gutiérrez, Pilar Alejandra Sáiz

**Affiliations:** 1 Department of Clinical Foundations, Pharmacology Unit, University of Barcelona, Barcelona, Spain; 2 Institut d’investigacions Biomèdiques August Pi i Sunyer (IDIBAPs), Barcelona, Spain; 3 Centro de Investigación Biomédica en red en salud Mental (CIBERSAM), Barcelona, Spain; 4 Barcelona Clínic Schizophrenia Unit (BCSU), Neuroscience Institute, Hospital Clínic de Barcelona, Barcelona, Spain; 5 Department of Psychiatry, Complejo Hospitalario de Navarra, Pamplona, Spain; 6 IdiSNA, Navarra Institute for Health Research, Pamplona, Spain; 7 Department of Child and Adolescent Psychiatry, Institute of Psychiatry and Mental Health, Hospital General Universitario Gregorio Marañón, IiSGM, School of Medicine, Universidad Complutense, Madrid, Spain; 8 Centro de Investigación Biomédica en Red de Salud Mental (CIBERSAM), Madrid, Spain; 9 Department of Psychiatry, Hospital Universitario de Alava, Vitoria, Spain; 10 BIOARABA Health Research Institute, Vitoria, Spain; 11 University of the Basque Country, Vitoria, Spain; 12 Hospital del Mar Medicar Research Institute (IMIM), Barcelona, Spain; 13 Autonomous University of Barcelona, Barcelona, Spain; 14 Instituto de Investigación Sanitaria Hospital 12 de Octubre (imas12), Madrid, Spain; 15 CogPsy Group, Universidad Complutense de Madrid (UCM), Madrid, Spain; 16 Psychiatry Department, Institut d’Investigació Biomèdica-Sant Pau (IIB-SANT PAU), Hospital de la Santa Creu i Sant Pau, Barcelona, Spain; 17 Universitat Autònoma de Barcelona (UAB), Barcelona, Spain; 18 FIDMAG Germanes Hospitalàries Research Foundation, Barcelona, Spain; 19 School of Medicine, Universitat Internacional de Catalunya, Barcelona, Spain; 20 Department of Psychiatry, Hospital Universitario Ramón y Cajal, IRYCIS, Universidad de Alcalá, Madrid, Spain; 21 Parc Sanitari Sant Joan de Déu, Institut de Recerca Sant Joan de Déu, Barcelona, Spain; 22 Hospital Sant Joan de Déu, Barcelona, Spain; 23 Bellvitge Biomedical Research Institute IDIBELL, Department of Psychiatry, Bellvitge University Hospital, Hospitalet de Llobregat- Barcelona, Barcelona, Spain; 24 Biomedical Research Networking Center for Mental Health Network (CIBERSAM), Barcelona, Spain; 25 Department of Medicine, University of Barcelona, Barcelona, Spain; 26 Neuropsychopharmacology and Psychobiology Research Group, Department of Psychology, University of Cádiz, Cádiz, Spain; 27 Instituto de Investigación e Innovación Biomédica de Cádiz, INiBICA, Hospital Universitario Puerta del Mar, Cádiz, Spain; 28 Cellex, IDIBAPS, University of Barcelona-Hospital Clínic of Barcelona, Barcelona, 08036 Spain; 29 Centro deInvestigación Biomédica en Red (CIBER) de Enfermedades Raras (CIBERER), Madrid, 28029 Spain; 30 Hospital del Mar Medical Research Institute (IMIM), Barcelona, Spain; 31 Autonomous University of Barcelona, Barcelona, Spain; 32 Instituto de Investigación Sanitaria Hospital 12 de Octubre (imas12), Madrid, Spain; 33 FIDMAG Germanes Hospitalàries Research Foundation, Barcelona, Spain; 34 Centro de Investigación Biomédica en Red de Salud Mental, CIBERSAM, Madrid, Spain; 35 Benito Menni CASM, Sant Boi de Llobregat, Spain; 36 Parc Sanitari Sant Joan de Déu, Sant Boi de Llobregat, Barcelona, Catalonia Spain; 37 Institut de Recerca Sant Joan de Déu, Esplugues de Llobregat, Barcelona, Spain; 38 Hospital Materno-Infantil Sant Joan de Déu, Esplugues de Llobregat, Barcelona, Catalonia Spain; 39 Fundación para la Investigación Biomédica del Hospital Universitario Ramón y Cajal (FIBioHRC), Hospital Universitario Ramón y Cajal, Madrid, Spain; 40 Neurobiology Unit, Program in Neurosciences and Institute of Biotechnology and Biomedicine (BIOTECMED), Universitat de València, Burjassot, Spain; 41 Biomedical Research Networking Centre in Mental Health (CIBERSAM), Madrid, Spain; 42 Biomedical Research Institute INCLIVA, Valencia, Spain; 43 Hospital Clínico Universitario and Instituto de Investigación Sanitaria (IIS) Aragón, Zaragoza, Spain; 44 Department of Medicine and Psychiatry, Universidad de Zaragoza, Zaragoza, Spain; 45 Centro de Investigación Biomédica en red en salud Mental (CIBERSAM), Barcelona, Spain; 46 Araba University Hospital, Bioaraba Research Institute, Vitoria, Spain; 47 Department of Psychiatry, School of Medicine, University of Oviedo, Instituto de Investigación Sanitaria del Principado de Asturias, Mental Health Services of Principado de Asturias, Biomedical Research Networking Centre in Mental Health (CIBERSAM), Oviedo, Spain

**Keywords:** Biomarkers, Psychosis

## Abstract

A better understanding of schizophrenia subtypes is necessary to stratify the patients according to clinical attributes. To explore the genomic architecture of schizophrenia symptomatology, we analyzed blood co-expression modules and their association with clinical data from patients in remission after a first episode of schizophrenia. In total, 91 participants of the 2EPS project were included. Gene expression was assessed using the Clariom S Human Array. Weighted-gene co-expression network analysis (WGCNA) was applied to identify modules of co-expressed genes and to test its correlation with global functioning, clinical symptomatology, and premorbid adjustment. Among the 25 modules identified, six modules were significantly correlated with clinical data. These modules could be clustered in two groups according to their correlation with clinical data. Hub genes in each group showing overlap with risk genes for schizophrenia were enriched in biological processes related to metabolic processes, regulation of gene expression, cellular localization and protein transport, immune processes, and neurotrophin pathways. Our results indicate that modules with significant associations with clinical data showed overlap with gene sets previously identified in differential gene-expression analysis in brain, indicating that peripheral tissues could reveal pathogenic mechanisms. Hub genes involved in these modules revealed multiple signaling pathways previously related to schizophrenia, which may represent the complex interplay in the pathological mechanisms behind the disease. These genes could represent potential targets for the development of peripheral biomarkers underlying illness traits in clinical remission stages after a first episode of schizophrenia.

## Introduction

Schizophrenia is a severe, lifelong mental disorder that can lead to significant functional impairment. It is a multifactorial disorder, where both genetic variants and environmental factors are important for its etiology^1,2^. The largest genetic studies of schizophrenia have highlighted its polygenic basis, with many genetic loci, mostly with small effect size^3^. Clinical symptomatology and response to antipsychotics, the cornerstone medication treatment for schizophrenia, varies largely between patients. Developing a better understanding of schizophrenia subtypes could contribute to the development of personalized medicine. For this, it is necessary to identify biomarkers to stratify the patients according to clinical attributes (i.e., the symptoms) that allow such medical care to be directed more effectively.

Within this setting, there is a need to understand how genomic variation could explain the existing heterogeneity in symptoms, treatment response, course, and outcome in schizophrenia, and to determine if genomic data could be a useful tool to define patients or groups of patients for clinical purposes. To understand how genomic variation contributes to schizophrenia heterogeneity and the biological insights behind clinical stratification, we have to move from genetic variants to genes, and from genes to networks^4^. Genes act in coordination, affecting the function of other genes in order to influence a particular phenotype through cellular pathways that are intertwined in complex networks. Network analysis could define disorder-associated molecular pathology and has allowed the identification of molecular pathways involved in schizophrenia risk^5,6^. Transcriptomic network analysis based on gene co-expression identified transcriptional networks dysregulated in schizophrenia and its cellular architecture^7,8^. Network analysis can interrogate multiple levels of molecular organization and provide a biological interpretation to clinical phenotypes^9^.

Therefore, to explore the genomic architecture of schizophrenia symptomatology, we analyzed blood co-expression modules, i.e., clusters of genes with highly correlated expression, in a cohort of remitted first-episode schizophrenia patients with <5 years of evolution, and their association with clinical data, including global functioning, clinical symptomatology, and premorbid adjustment.

## Results

### WGCNA results and validation of the gene co-expression network

Table 1 shows the demographic, clinical, and pharmacological characteristics of the 91 participants in the present study. One sample with connectivity less than −5 was removed (Supplementary Fig. 1A). We identified 24 modules of co-expressed genes (Supplementary Fig. 1C). The inferred modules showed different sizes ranging from 41 (Dark gray module) to 5627 genes (Turquoise module). A further 1901 genes were assigned into the Gray module, which represents the genes that were not co-expressed based on gene dissimilarity. Importantly, the organization of our co-expression modules was robustly defined in our cohort (Supplementary Fig. 2) and significantly overlapped with the modules originally reported in peripheral blood described by Gudmundsdottir et al.^10^ (Supplementary Table 1). 22 modules of the 24 identified in our analysis (excluding the Gray module) showed significant overlap with 41 of the 55 modules identified by Gudmundsdottir et al.^10^.

**Table 1:** Demographic, clinical and pharmacological data of the 91 participants in the present study.

	Baseline
*N*	91
Age, mean ± SD	25.3 ± 5.85
Age at first diagnosis, mean ± SD	24.1 ± 5.7
Gender, male, *N* (%)	62 (67.4)
Ethnicity, Caucasian, *N* (%)	81 (88.0)
Functioning	
CGI (mean ± SD)	3.2 ± 1.2
GAF (mean ± SD)	69 ± 14.5
FAST (mean ± SD)	20.2 ± 15.7
Psychotic symptoms	
PANSS positive (mean ± SD)	9.6 ± 3.2
PANSS negative (mean ± SD)	13.9 ± 5.4
PANSS general (mean ± SD)	25.6 ± 7.9
PANSS total (mean ± SD)	49.1 ± 14.8
Affective symptoms	
YMRS (mean ± SD)	0.8 ± 1.5
MADRS (mean ± SD)	6.9 ± 6.3
PAS (mean ± SD)	45.7 ± 21.4
Antipsychotic	
Aripiprazol, *N* (%)	34 (40.5)
Paliperidone, *N* (%)	24 (28.6)
Risperidone, *N* (%)	13 (15.4)
Olanzapine, *N* (%)	12 (14.3)
Clozapine, *N* (%)	6 (7.1)
Quetiapine, *N* (%)	3 (3.6)
Amisulpride, *N* (%)	3 (3.6)
Clotiapine, *N* (%)	2 (2.4)
Asenapine, *N* (%)	1 (1.2)
Haloperidol, *N* (%)	1 (1.2)
Ziprasidone, *N* (%)	1 (1.2)
Co-medication	
Antidepressant, Yes, N (%)	24 (28.6)
Anxiolytic, Yes, N (%)	13 (15.5)
Lithium, Yes, N (%)	2 (2.4)
Antiepileptic, Yes, N (%)	5 (5.9)
Antiparkinsonian, Yes, N (%)	11 (13.1)

### Identification of co-expression networks related to clinical data

**Fig. 1:**
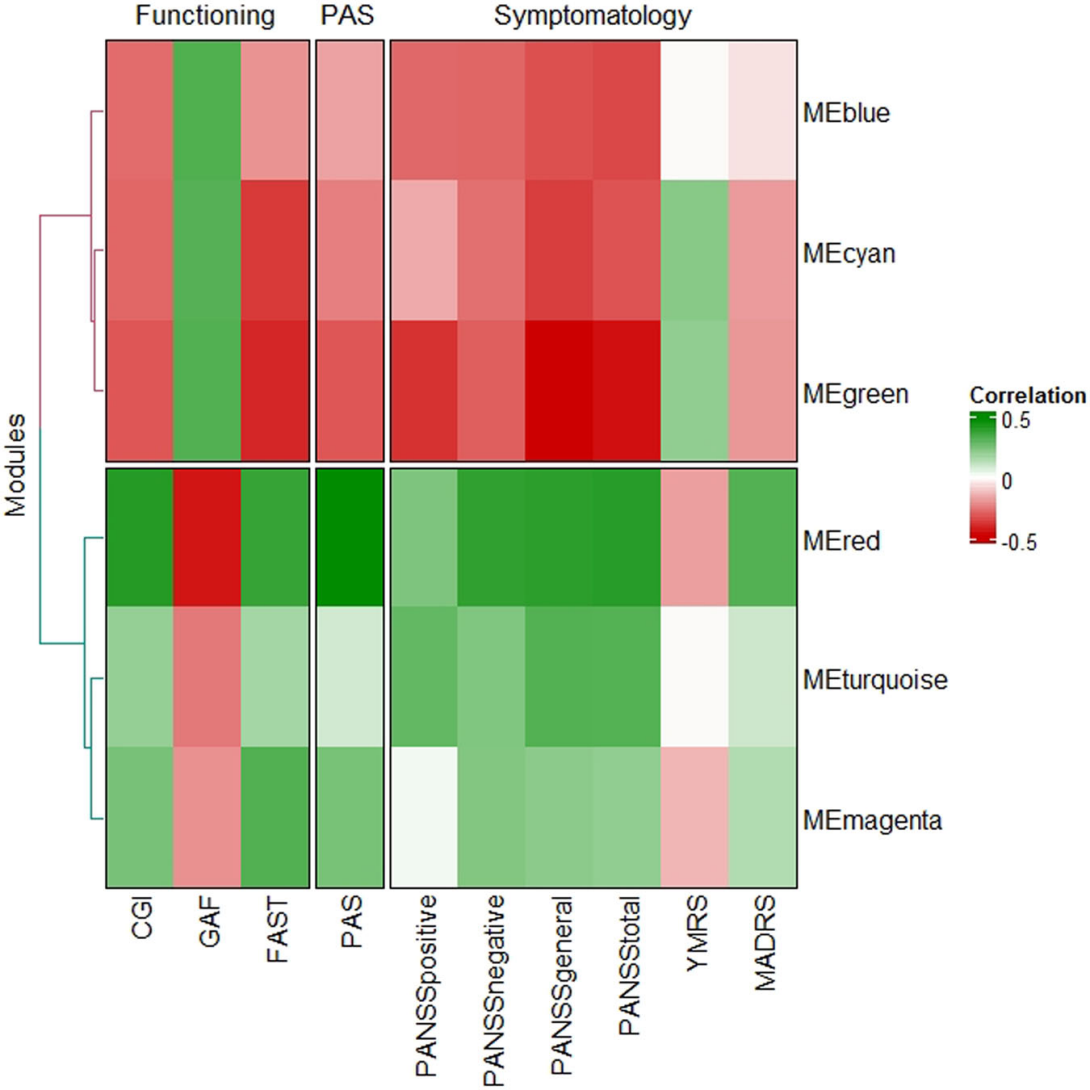
Heatmap of the Pearson correlation coefficient between module eigengenes (MEs) and clinical information. The color of the cell reflects the size of the correlation coefficient, as shown in the legend on the right.

We found that MEs of six modules were significantly correlated with clinical data after correcting for multiple testing (*p* < 0.007) (Table 2 and Supplementary Table 2). These modules could be grouped according to their correlations with global functioning, clinical symptomatology, and premorbid adjustment in two clusters (Fig. 1). Cluster 1 included the Blue, Green, and Cyan modules, showing positive correlations with global functioning measured with the CGI, GAF, and FAST scores, negative correlations with psychotic symptoms measured with the PANSS scale and its subscales (positive, negative, and general), and positive correlations with premorbid adjustment measured with the PAS scale (the Blue module was not significant for the latter). Cluster 2 was formed by the Red, Turquoise, and Magenta modules, which showed an opposite pattern to Cluster 1 modules, with negative correlations with global functioning, positive correlations with psychotic symptoms, and negative correlations with premorbid adjustment.

**Table 2:** Correlation coefficients between the module eigenvalues and clinical variables of the six modules showing significant correlations after multiple testing corrections (*p* < 0.007).

	Functionality			Symptomatology						PAS
Module	CGI	GAF	FAST	PANSS positive	PANSS negative	PANSS general	PANSS total	YMRS	MADRS	PAS
MEturquoise	0.190	−0.255*	0.162	0.283*	0.229*	0.313**	0.311**	−0.011	0.088	0.081
MEmagenta	0.244*	−0.211	0.315**	0.017	0.226*	0.209	0.197	−0.141	0.138	0.243*
MEcyan	−0.291*	0.308**	−0.374**	−0.164	−0.273*	−0.366**	−0.329**	0.214	−0.192	−0.243*
MEblue	−0.283*	0.320**	−0.207	−0.287*	−0.294**	−0.332**	−0.346**	−0.011	−0.061	−0.179
MEgreen	−0.320**	0.315**	−0.411**	−0.389**	−0.306**	−0.482**	−0.451**	0.198	−0.200	−0.320**
MEred	0.399**	−0.442**	0.370**	0.232**	0.380**	0.387**	0.394**	−0.186	0.313**	0.469**

*p < 0.05; **p < 0.007.

*CGI* Clinical Global Impression Scale, *GAF* Global Assessment of Functioning Scale, *FAST* Functional Assessment Staging Test, *PANSS* Positive and Negative Syndrome Scale, *YMRS* Young Mania Rating Scale, *MADRS* Montgomery–Asberg Depression Rating Scale, *PAS* Premorbid Adjustment Scale.

Five additional modules (Yellow, Black Grey60, Cyan, and Darkgreen) showed significant correlations with some clinical variables. However, these modules did not show a significant correlation between GS and MM. The lack of a GS–MM correlation could indicate that only a submodule relates to the trait or suggests that the association should be considered as more tentative, needing further validation or evidence. For this reason, these modules were not selected for further analysis.

### Identification of hub genes and external validation of relevant modules

For each module, “hub genes" (more centralized genes in the network) were defined according to their module membership (MM > 0.8) and their gene significance (GS > 0.3) (Supplementary Table 3). Hub genes from each module were combined with the hub genes of the other modules included in the same defined cluster and used to create gene sets. These gene sets were tested for overlap with gene sets previously associated with schizophrenia in gene expression studies in brain samples. The 1331 hub genes from Cluster 1 showed significant overlap with genes involved in several modules identified in DLPFC of schizophrenia patients in the studies of Fromer et al.^7^ (five modules) and Gandal et al.^8^ (one module) (Table 3). Among these modules, M11 and M13 modules from Fromer et al.^7^ were significantly associated with schizophrenia in the original study. The 403 hub genes from Cluster 2 showed significant overlap with four modules reported by Fromer et al.^7^ and four models reported by Gandal et al.^8^. Among these modules, M2 from Fromer et al.^7^ was significantly associated with schizophrenia in the original study but also significantly enriched with genes associated with schizophrenia in genetic studies that include genome-wide association studies but also copy number variants and rare variants studies^7^. The Turquoise module from Gandal et al.^8^ was significantly associated with schizophrenia risk. Moreover, the M2 and the Turquoise modules were enriched with neuronal cell markers^7,8^.

**Table 3:** Gene overlap between hub genes from module clusters and modules reported by Fromer et al.^7^ and Gandal et al.^8^.

	Study	Module	Overlapping genes	Corrected *P*-values
Cluster 1	Fromer	M10s	42	0.049
	Fromer	M11s	94	1.30 × 10^−7^
	Fromer	M13s	81	3.49 × 10^−7^
	Fromer	M6s	58	0.001
	Fromer	M8s	111	1.02 × 10^−10^
	Gandal	Green	158	2.28 × 10^−8^
Cluster 2	Fromer	M1s	39	2.65 × 10^−15^
	Fromer	M2s	24	1.08 × 10^−9^
	Fromer	M3s	17	0.002
	Fromer	M4s	18	7.90 × 10^−10^
	Gandal	Blue	43	6.04 × 10^−21^
	Gandal	Pink	12	3.80 × 10^−7^
	Gandal	Red	20	5.56 × 10^−7^
	Gandal	Turquoise	32	4.77 × 10^−13^

Table shows the module, the number of overlapping genes, and the corrected *p*-value of hypergeometric test statistics.

**Fig. 2:**
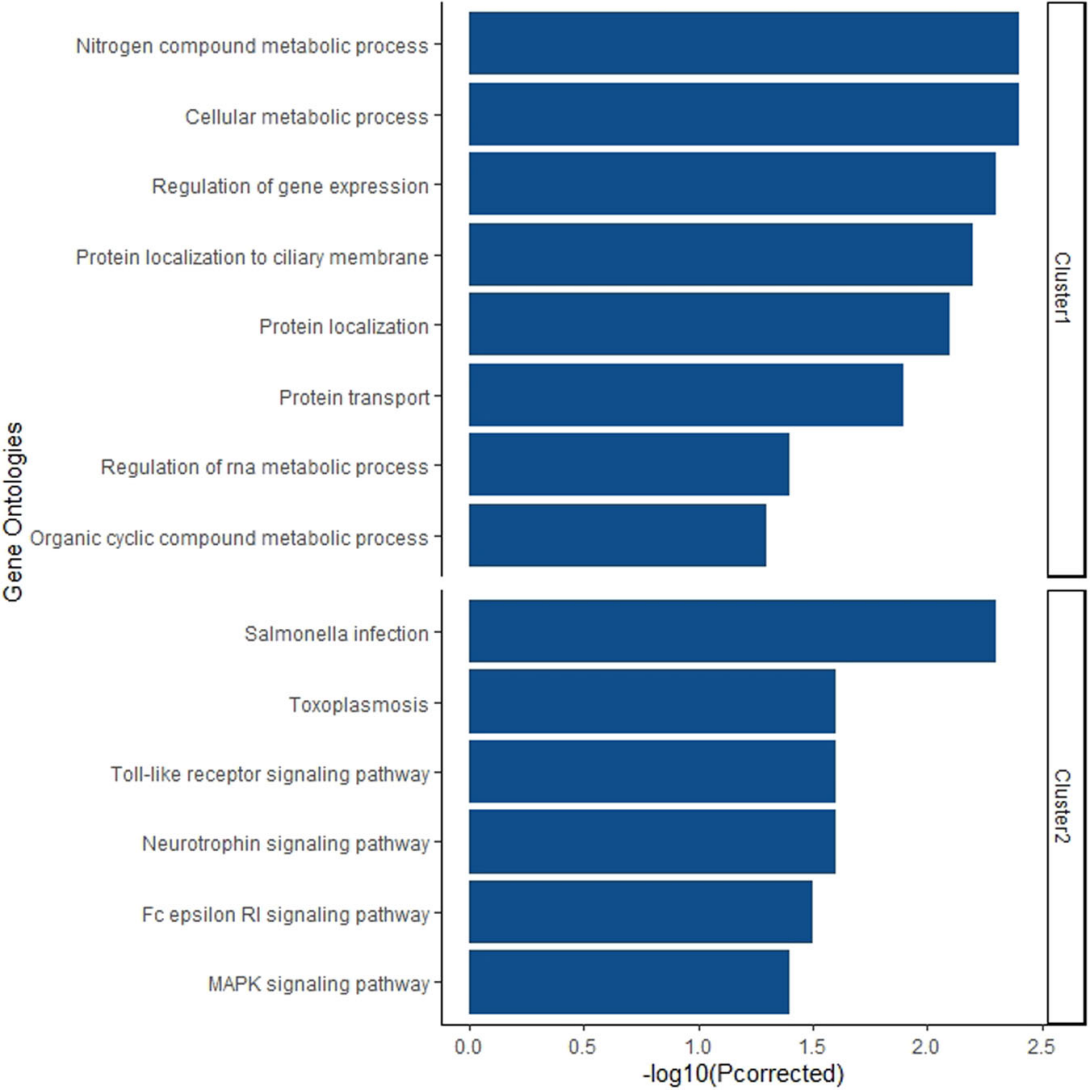
Gene-set enrichment analysis (Gene Ontology Biological Process) of each Cluster. Only significant terms are shown (adjusted *p*-value < 0.05).

Finally, the gene set enrichment analysis of the hub gene sets defined for each cluster is shown in Fig. 2.

## Discussion

We performed an analysis of gene co-expression architecture in peripheral blood in a cohort of remitted first-episode schizophrenia patients with <5 years of evolution. We identified six modules of co-expressed genes with significant associations with clinical data that showed significant overlap with genes previously associated with schizophrenia in brain samples. These modules were clustered into two groups according to their association and the direction of these associations, and the hub genes of each cluster were enriched with functional ontologies. The purpose of the study was to identify and prioritize co-expressed gene sets based on their association with clinical data and their overlap with genes previously associated with schizophrenia in large datasets. The purpose of the study was to identify and prioritize co-expressed gene sets based on their association with clinical data and their overlap with genes previously associated with schizophrenia in large datasets, in order to identify peripherial biomarkers that could ultimately translate into the clinical practice with the stratification of patients according to the underlying illness traits in clinical remission stages after a first-episode schizophrenia.

We clustered modules with significant correlations with clinical variables to provide a more clinical interpretation of our results. Some modules showed significant associations with the same clinical variables and directions, although we could not detect a strong correlation among them. These modules could include genes involved in similar biological processes that could explain its effect on patients’ functionality, symptomatology, and premorbid adjustment, although these genes were clustered in different co-expression modules. The functional analysis and interpretation were focused on the hub genes of these clusters. There is an ongoing debate in the literature regarding the importance of hub genes, central nodes in network architecture, and its importance in functional analysis. On this regard, several authors reported that intramodular hub genes from coexpression networks instead of whole network hubs are more significantly related to a trait and more often of clinical importance^11,12^. Moreover, selecting intramodular hubs in relevant modules often leads to gene lists with cleaner biological annotations after functional enrichment analysis evaluation^13^. This is relevant for studying candidate biological processes associated with the trait of interest.

Lower expression of genes in Cluster 1 correlated with poor global functioning, the worst severity of psychotic symptoms, and low premorbid adjustment. The hub genes of this group participated in the chemical reactions and pathways involving compounds such as catecholamine (norepinephrine and dopamine) and serotonin, the neurotransmitters that have been classically related to schizophrenia psychopathology^14^. The presence of multiple biological processes and signaling pathways in the module, such as the metabolism of neurotransmitters and the regulation of gene expression, and the regulation and transport of proteins in the cell, could reflect complex interplays between these processes in the neurobiology of the disease. This crosstalk between signaling pathways is a critical issue in the underlying polygenic architecture of complex diseases such as schizophrenia^15^. Among these genes, there are several genes that have been widely studied as candidate genes involved in schizophrenia, especially Sirtuin 1 (*SIRT1*)^16–21^. Other genes such as the ataxia-telangiectasia mutated serine/threonine kinase (*ATM*)^22,23^, the ubiquitin-like modifier activating enzyme 3 (*UBA3*)^24^, the neuroblastoma RAS proto-oncogene GTPase (*NRAS*)^25^, the cytotoxic granule associated RNA binding protein (*TIA1*)^26^, the splicing factor 3b subunit 1 (*SF3B1*)^27^, and the ubiquitin-conjugating enzyme E2 N (*UBE2N*)^28^ were also included in Cluster 1.

Genes belonging to modules included in Cluster 2 showed a significant correlation between higher gene expression and low functioning, higher severity of psychotic symptoms, and poor premorbid adjustment. Hub genes of this cluster, in contrast to cluster 1, were enriched mainly with biological processes related to inflammatory pathways. This is especially relevant considering the immunological and inflammatory hypothesis of schizophrenia and the possible role of inflammatory markers on symptoms severity^29^. Among enriched processes, two signaling pathways have been repeatedly proposed to be involved in the neurobiology of schizophrenia: the Toll-like receptor signaling pathway^30^ and the neurotrophin pathway^31^. Both biological processes have been shown to play a vital role in neuronal growth and differentiation, as well as in neuronal survival, synaptogenesis, and neuroplasticity in the adult brain^32,33^. Complex crosstalk between these processes has been proposed and could form the neurobiological basis of schizophrenia through the cellular communication between astrocytes and microglia^34–36^. A notable gene in the network is furin, a paired basic amino acid cleaving enzyme (*FURIN*), one of the loci that showed a significant overlap between GWAS for schizophrenia and eQTL in the DLPFC reported by Fromer et al.^7^. Moreover, in the same study, experimental suppression of this gene had an impact on neuroanatomical and developmental attributes in an experimental model of zebrafish neurodevelopment^7^. The involvement of *FURIN* in schizophrenia has been extensively studied in recent years^37–41^. One *FURIN* substrate is the von Willebrand factor (*VWF*), a critical gene in the Cluster 2. The VWF is a well-known marker of endothelial cell activation and inflammation that has been associated with brain morphology, cognitive functions, and affective and psychotic symptoms^42–45^. Other critical genes in the network included the MAP kinase kinase 7 (*MAP2K7*) related to functional plasticity in the brain and cognition^46–49^ and the forkhead box O3 (*FOXO3*), a transcription factor with numerous functions in neurodevelopment and adult brain that has been implicated in schizophrenia^50^.

Few studies analyzing gene expression in the peripheral blood have focused specifically on the transcriptome of first-episode psychosis, and its relation with clinical symptomatology, with the notable exception of the study of Leirer et al.^51^, although they use individual gene analysis.

Some limitations should be considered in the interpretation of our results. First, the sample size may have limited the statistical power of our analysis. Second, although modules of co-expressed genes provide potential insights to understand biological mechanisms, confirmatory evidence requires experimental studies. Last, due to the naturalistic design, drug treatment was not controlled, and the study participants maintained their usual treatment. Despite these limitations, the strength of this study lies in the inclusion of a consistent well-characterized first-episode schizophrenia-patient sample in remission and well-described symptoms and functioning.

In conclusion, our study offers a characterization of the cotranscriptome in the peripheral blood of a sample of first-episode schizophrenia patients in remission and its relation to global functioning, clinical symptomatology, and premorbid adjustment. Our results indicate that modules with significant associations with clinical data showed overlap with gene sets identified in differential gene-expression analysis in DLPFC, indicating that peripheral tissues could reveal pathogenic mechanisms. Hub genes involved in these modules revealed multiple signaling pathways, previously related to schizophrenia, which may represent the complex interplay in the pathological mechanisms behind the disease. Overall, Cluster 1 and 2 shared clinical correlates, although in opposite directions. This could be of great clinical interest. Critical genes in the functional networks could represent potential targets for the development of peripheral biomarkers underlying illness traits in clinical remission stages after a first episode of schizophrenia.

## Methods

### Study design

This study is part of a naturalistic, multicentre, coordinated, and multimodal project “Clinical and neurobiological determinants of second episodes of schizophrenia. Longitudinal study of first episode of psychosis” (PI11/00325), also known as the 2EPs Project^52^. The project includes multiple sub-studies: general and basic, neuroimaging, adherence, neurocognition, physical health, and biological. Given its main goals, the present study was framed within the general and biological modules.

### Subjects

The inclusion criteria for the 2EPs Project were (a) age between 16 and 40 years at the time of first assessment (baseline visit); (b) meeting diagnostic criteria according to DSM-IV for schizophrenia or schizophreniform disorder^53^; (c) being in remission from the first psychotic episode (which should have occurred within the last 5 years), according to Andreassen’s criteria^54^; (d) not having relapsed after the first psychotic episode; (e) speaking Spanish fluently; and (f) providing the signed informed consent form. The exclusion criteria were (a) having experienced a traumatic brain injury with loss of consciousness; (b) presenting intellectual disability understood not only as IQ <70, but also presenting malfunctioning and problems with adaptive processes; and/or (c) presenting somatic pathology with mental repercussion.

From the initial 223 patients recruited in the 2EPs Project, 91 (40.8%) participated in the biological module and provided a biological sample for gene expression analysis at baseline.

The study was approved by the investigation ethics committee of the Hospital Clinic (Barcelona, Spain). Informed consent was obtained from all participants. For children under the age of 18 years old, parents or legal guardians gave written informed consent before beginning their participation in the study, and patients assented to participate. This study was conducted in accordance with the Declaration of Helsinki.

### Clinical assessment

Demographic data were collected for all patients through semi-structured interviews. Diagnoses were determined according to the DSM-IV-criteria^53^, with the SCID-I^55^ or the Kiddie-SADS^56^ depending on age.

In order to obtain global functional outcome information, three different scales were used:

1. The Clinical Global Impression Scale (CGI-S)^57^ assesses the severity of global symptomatology;
2. The Functional Assessment Staging Test (FAST)^58^ evaluates the patient’s degree of difficulty in autonomy, work functioning, cognitive functioning, finance, interpersonal relationships, and free time functioning;
3. The Global Assessment of Functioning Scale (GAF)^59^ measures the severity of symptoms and the level of functioning.

The clinical symptomatology was assessed, separating by different areas as follows:

1. Psychotic symptoms were assessed using the Spanish validated version of the Positive and Negative Syndrome Scale (PANSS)^60^ which comprises three subscales (positive, negative, and general);
2. Affective symptoms were assessed using the Spanish validated version of the Young Mania Rating Scale (YMRS), designed to assess the severity of manic symptoms^61^ and the Spanish validated version of the Montgomery–Asberg Depression Rating Scale (MADRS), to assess the severity of depression^62^.

Finally, premorbid adjustment was assessed using the Premorbid Adjustment Scale (PAS)^63^ which explores sociability and withdrawal, peer relationships, school achievement, adaptation to school, and ability to establish socio-affective and sexual relationships.

### Sample collection, RNA isolation, and microarray hybridization

Peripheral blood was collected at baseline in PAXgene Blood RNA tubes and total RNA was isolated (PAXgene Blood RNA kit, PreAnalytiX Gmbh, Switzerland). The purity and integrity of RNA were assessed using an Agilent 2100 Bioanalyzer (Agilent Technologies, Palo Alto, CA, USA). The mean RNA integrity number (RIN) was 7.77 ± 0.70, ranging from 6.5 to 8.7. No sample was discarded due to low RIN number. A total of 1 μg of purified RNA from each of the samples was submitted to the Kompetenzzentrum für Fluoreszente Bioanalytik Microarray Technology (KFB, BioPark Regensburg GmbH, Regensburg, Germany) for labeling and hybridization to the Clariom S Human Array (Affymetrix, Santa Clara, CA, USA), following the manufacturer’s protocols. The Clariom S Human Array comprises more than 221,300 probes covering over 337,100 transcripts and variants, which in turn represent 20,800 genes.

### Genome-wide expression analysis and the WGCNA procedure

Microarray data preprocessing was performed using the Oligo R package^64^. The data were standardized using robust multichip analysis. Multiple probes mapping to the same gene were merged using the average as the summary of the hybridization values. Normalized expression data from all samples were used to identify and remove any source of unwanted variation before the construction of co-expression networks. We applied the *sva* R package that contains functions for identifying and building surrogate variables for high-dimensional data^65^. First, the normalized data matrix was converted in an *ExpressionSet* object. Second, we applied the *sva* function using the *n.sv* argument and the *num.sv* function to estimate the number of surrogate variables. After applying this approach, the *sva* functions returned 0 arguments, assuming that no-sources of unwanted variation are present in our gene expression matrix. Cell counts were measured using the Gene Expression Deconvolution Interactive Tool (GEDIT)^66^. The following cells were counted: CD8+ T cells, CD4+ T cells, natural killer (NK) cells, B cells, and monocytes, using the Human Body atlas as reference matrix^67^.

Co-expression modules were identified using the R software package for weighted gene co-expression network analysis (WGCNA)^68^. First, in order to remove outlier samples, distance-based adjacency matrices of samples were estimated and sample network connectivity according to the distances was standardized. Samples with connectivity less than –5 were considered as outliers and were excluded (Supplementary Fig. 1A). The co-expression analysis involved constructing a matrix of pairwise correlations between all pairs of genes across all selected samples. Next, the matrix was raised to a soft-thresholding power (*β* = 6 in this study) to obtain an adjacency matrix (Supplementary Fig. 1B). To identify modules of co-expressed genes, we constructed the topological overlap-based dissimilarity, which was then used as input to average linkage hierarchical clustering. This step resulted in a clustering tree (dendrogram), branches of which were identified for cutting based on their shape, using the dynamic tree-cutting algorithm (Supplementary Fig. 1C). The above steps were performed using the automatic network construction and module detection function (*blockwiseModules* in WGCNA), with the following parameters: minModuleSize of 30, reassignThreshold of 0, and mergeCutHeight of 0.25.

The modules were then tested for their associations with clinical variables by correlating module eigengenes (MEs, defined as the first principal component of each module) with scale values using Pearson partial correlation. For each significant module, the correlation between the gene significance (GS, the absolute value of the Pearson correlation between each gene expression and scale score) and its module membership (MM, the correlation between gene expression and the module eigengene) was calculated adjusted by sex, age, age at onset, ethnicity, and cell count. Boostrapping was used as a cross-validation approach and the confidence intervals for the correlation coefficient were estimated using 1000 random samples. Multiple testing corrections were applied using the false discovery rate (FDR) as described by Benjamini & Hochberg. According to this method, *p*-value < 0.007 was considered statistically significant.

Modules showing significant correlations with clinical data after multiple testing corrections were clustered according to the computed correlation coefficients. We first calculated the distance matrix and then performed hierarchical clustering of modules as implemented in the *ComplexHeatmap* R package^69^.

### Validation of the gene co-expression network

To assess whether the resulting co-expression modules were robustly defined in our cohort, we performed a subsampling analysis (Supplementary Fig. 2). This analysis consisted in the network construction and module identification using the previous parameters, with 50 iterations including randomly drawn individuals, as implemented in the sampleBlockwiseModules function in the WGCNA R package. For each gene, the consistency was calculated as the percentage of iterations in which it was assigned to the original module. Finally, the stability of each module was defined as the average gene consistency of all genes constituting the given module.

An external validation was performed assessing the replication of the identified co-expression modules in the general blood architecture [of an independent sample]. To this end, we test the degree of overlap, using the hypergeometric test implemented in the *userListEnrichment* function from the WGCNA package, between our modules and the modules described in peripheral blood in a large cohort of healthy human samples (*N* = 2127)^10^.

### External validation of relevant modules

To characterize modules significantly associated with clinical symptomatology, the hub genes in each module were used to define a gene set. Hub genes were defined according to their gene significance (GS) with the clinical variable of interest (GS > 0.3) and their module membership (MM > 0.8). These gene sets were tested for overlap with the gene composition and module labels from gene expression studies in dorsolateral prefrontal cortex (DLPFC) of large cohorts of schizophrenia subjects, including studies from Fromer et al. (*N* = 159 schizophrenia patients)^7^ and Gandal et al.^8^ (*N* = 258 schizophrenia patients)^8^. We also compared our list of hub genes with genes associated with schizophrenia using gene expression imputation (Transcriptome Wide Analysis, TWAS) across multiple brain regions in 40,299 schizophrenia cases and 65,264 matched controls^70^. To test the degree of overlap, we used the hypergeometric test implemented in the *userListEnrichment* function from the WGCNA package.

The gene sets of selected hub genes were imported to ClueGO v2.1^71^, to perform a gene set enrichment analysis. The Biological Processes of the Gene Ontology databases were selected for the enrichment analysis. Genes involved in each network were mapped to their enriched Biological Processes based on the hypergeometric test (two-sided), with the *p*-value being corrected using the Benjamini–Hochberg method (adjusted *P* < 0.05 was considered significant).

## Supplementary information

Supplementary Information

## Data Availability

Gene expression data that support the findings of this study have been deposited in Gene Expression Omnibus (GEO) Database (https://www.ncbi.nlm.nih.gov/geo/) with the accession code GSE193818. The clinical data that support the findings of this study are not openly available due to contain human data and are available from the corresponding author upon reasonable request.
