# Supplementary material for: Gene co-expression architecture in peripheral blood in a cohort of remitted first-episode schizophrenia patients
Source: Schizophrenia (Heidelb). 2022 Apr 27;8(1):45. doi: 10.1038/s41537-022-00215-1 (PMC9261105; doi:10.1038/s41537-022-00215-1)

**Supplementary Table 1.** Comparison between our modules and modules reported in Gudmundsdottir et al., 2020 (p values of hypergeometric test statistics and overlapping genes)

| **Modules** | **Gudmundsdottir et al., 2020 Modules** | **CorrectedPvalues** |
| --- | --- | --- |
| blue | Blood_M3 | 1.79815466933949e-115 |
| grey60 | Blood_M15 | 4.52921374881392e-110 |
| lightcyan | Blood_M29 | 1.73154473615676e-95 |
| lightyellow | Blood_M18 | 7.53713161779445e-93 |
| magenta | Blood_M9 | 1.42264478325225e-82 |
| red | Blood_M30 | 1.30445997930807e-75 |
| greenyellow | Blood_M8 | 6.16008172274099e-75 |
| darkturquoise | Blood_M17 | 2.26885649256782e-73 |
| blue | Blood_M1 | 9.45717644958615e-68 |
| magenta | Blood_M8 | 1.3257907379457e-64 |
| purple | Blood_M8 | 7.64207823706669e-64 |
| blue | Blood_M10 | 1.92882487558193e-45 |
| black | Blood_M2 | 1.3653528783518e-43 |
| grey | Blood_M0 | 1.96134970370687e-39 |
| blue | Blood_M12 | 1.56470358578138e-34 |
| lightcyan | Blood_M32 | 3.00533054170209e-28 |
| magenta | Blood_M14 | 9.33945031635092e-27 |
| tan | Blood_M2 | 4.9486406416648e-25 |
| greenyellow | Blood_M9 | 1.59415388787691e-24 |
| turquoise | Blood_M0 | 4.35641568085813e-21 |
| midnightblue | Blood_M14 | 9.56559314740015e-21 |
| grey60 | Blood_M46 | 1.31114703057662e-15 |
| cyan | Blood_M13 | 3.06256026491694e-15 |
| lightgreen | Blood_M14 | 6.7766705136179e-13 |
| purple | Blood_M39 | 1.00610918864411e-11 |
| royalblue | Blood_M2 | 1.04683547333483e-11 |
| salmon | Blood_M13 | 5.98289771903362e-11 |
| tan | Blood_M7 | 1.942086789076e-10 |
| green | Blood_M1 | 2.90216888669361e-10 |
| magenta | Blood_M36 | 3.93572032395631e-10 |
| black | Blood_M51 | 1.0553792512203e-09 |
| blue | Blood_M26 | 2.74691593016739e-09 |
| greenyellow | Blood_M14 | 5.92544209520539e-09 |
| tan | Blood_M6 | 6.57510918075261e-09 |
| blue | Blood_M19 | 1.82909147587661e-08 |
| darkgrey | Blood_M2 | 4.08945332657814e-08 |
| blue | Blood_M20 | 5.38844391655663e-08 |
| royalblue | Blood_M7 | 8.01328978938718e-08 |
| lightcyan | Blood_M50 | 2.52856480153476e-07 |
| blue | Blood_M24 | 8.86835406661218e-07 |
| salmon | Blood_M1 | 1.32727971209422e-06 |
| black | Blood_M7 | 1.48382920529521e-06 |
| yellow | Blood_M14 | 1.55531657922454e-06 |
| green | Blood_M10 | 3.90302811467397e-06 |
| salmon | Blood_M2 | 5.1271813449977e-06 |
| yellow | Blood_M16 | 8.98959548116202e-06 |
| lightgreen | Blood_M9 | 9.66221879154815e-06 |
| grey | Blood_M35 | 1.97045994717642e-05 |
| grey | Blood_M52 | 3.73206340564566e-05 |
| yellow | Blood_M42 | 4.69293750847643e-05 |
| darkgrey | Blood_M40 | 0.000134199203522765 |
| greenyellow | Blood_M43 | 0.000179195400298236 |
| lightgreen | Blood_M25 | 0.000205812048503634 |
| purple | Blood_M19 | 0.000238712467317628 |
| midnightblue | Blood_M7 | 0.0009874869221957 |
| red | Blood_M0 | 0.00136692727646452 |
| green | Blood_M3 | 0.00166674020961942 |
| grey | Blood_M34 | 0.00234352573129613 |
| purple | Blood_M9 | 0.00246011526081612 |
| lightcyan | Blood_M22 | 0.00275422157473988 |
| purple | Blood_M36 | 0.00317849261374188 |
| yellow | Blood_M37 | 0.00323172209044108 |
| yellow | Blood_M11 | 0.00480419093404625 |
| greenyellow | Blood_M19 | 0.00560714195126272 |
| red | Blood_M18 | 0.00911916491041117 |
| pink | Blood_M31 | 0.00982965218590864 |
| blue | Blood_M23 | 0.0139958555448293 |
| greenyellow | Blood_M39 | 0.0160189513055197 |
| grey | Blood_M28 | 0.016340643134034 |
| lightcyan | Blood_M39 | 0.026227726878869 |
| yellow | Blood_M5 | 0.0385197796372375 |
| black | Blood_M40 | 0.0434692367663461 |
| greenyellow | Blood_M25 | 0.0439157838526785 |
| magenta | Blood_M39 | 0.0488562438178488 |

**Supplementary Table 2.**  Module eigenvalues and clinical variables of all modules. Table shows the correlation coefficient, the p-value and the 95% confidence intervals estimated by boostrapping.

|  |  |  | **Functioning** | | **Symptomatology** | | | | | | **PAS** |
| --- | --- | --- | --- | --- | --- | --- | --- | --- | --- | --- | --- |
|  |  |  | **CGI** | **FAST** | **PANSS Positive** | **PANSS Negative** | **PANSS General** | **PANSS Total** | **YMRS** | **MADRS** | **PAS** |
| **MEdarkred** | Coefficient |  | 0.017 | -0.060 | 0.056 | -0.018 | 0.091 | 0.053 | 0.232 | 0.014 | -0.153 |
|  | p-value |  | 0.883 | 0.601 | 0.629 | 0.873 | 0.427 | 0.643 | 0.041 | 0.902 | 0.181 |
|  | 95 % CI | Lower | -00.175 | -00.233 | -00.112 | -00.209 | -00.085 | -00.116 | -00.086 | -00.155 | -00.32 |
|  |  | Upper | 00.239 | 00.125 | 00.207 | 00.191 | 00.258 | 00.229 | 00.484 | 00.197 | 00.051 |
| **MEbrown** | Coefficient |  | 0.149 | -0.035 | 0.110 | 0.116 | 0.057 | 0.097 | 0.261 | -0.030 | -0.065 |
|  | p-value |  | 0.194 | 0.761 | 0.339 | 0.310 | 0.618 | 0.397 | 0.021 | 0.795 | 0.570 |
|  | 95 % CI | Lower | -00.023 | -00.216 | -00.086 | -00.074 | -00.143 | -00.083 | -00.167 | -00.19 | -00.238 |
|  |  | Upper | 00.334 | 00.168 | 00.287 | 00.315 | 00.294 | 00.29 | 00.567 | 00.168 | 00.142 |
| **MEturquoise** | Coefficient |  | 0.190 | 0.162 | 0.283 | 0.229 | 0.313 | 0.311 | -0.011 | 0.088 | 0.081 |
|  | p-value |  | 0.096 | 0.157 | 0.012 | 0.044 | 0.005 | 0.006 | 0.926 | 0.443 | 0.482 |
|  | 95 % CI | Lower | 00.022 | -00.041 | 00.101 | 00.011 | 00.114 | 00.121 | -00.322 | -00.098 | -00.115 |
|  |  | Upper | 00.371 | 00.381 | 00.467 | 00.439 | 00.49 | 00.489 | 00.247 | 00.301 | 00.302 |
| **MElightgreen** | Coefficient |  | 0.282 | 0.211 | 0.109 | 0.210 | 0.209 | 0.211 | -0.090 | 0.027 | 0.162 |
|  | p-value |  | 0.012 | 0.064 | 0.344 | 0.065 | 0.066 | 0.063 | 0.433 | 0.816 | 0.157 |
|  | 95 % CI | Lower | 00.116 | -00.015 | -00.064 | 00.006 | -00.011 | -00.005 | -00.337 | -00.171 | -00.073 |
|  |  | Upper | 00.453 | 00.448 | 00.273 | 00.399 | 00.429 | 00.416 | 00.136 | 00.268 | 00.407 |
| **MEmagenta** | Coefficient |  | 0.244 | 0.315 | 0.017 | 0.226 | 0.209 | 0.197 | -0.141 | 0.138 | 0.243 |
|  | p-value |  | 0.031 | 0.005 | 0.882 | 0.046 | 0.067 | 0.084 | 0.220 | 0.227 | 0.032 |
|  | 95 % CI | Lower | 00.028 | 00.095 | -00.193 | 00.014 | 00.008 | -00.004 | -00.311 | -00.08 | 00.025 |
|  |  | Upper | 00.454 | 00.524 | 00.204 | 00.445 | 00.4 | 00.401 | 00.032 | 00.372 | 00.45 |
| **MElightcyan** | Coefficient |  | 0.012 | 0.158 | -0.109 | -0.006 | 0.003 | -0.024 | -0.185 | 0.033 | 0.131 |
|  | p-value |  | 0.916 | 0.166 | 0.344 | 0.959 | 0.977 | 0.832 | 0.104 | 0.774 | 0.253 |
|  | 95 % CI | Lower | -00.153 | -00.006 | -00.275 | -00.165 | -00.153 | -00.182 | -00.396 | -00.122 | -00.077 |
|  |  | Upper | 00.165 | 00.307 | 00.081 | 00.159 | 00.196 | 00.152 | 00.054 | 00.225 | 00.338 |
| **MEgreenyellow** | Coefficient |  | 0.168 | 0.334 | -0.049 | 0.142 | 0.180 | 0.136 | -0.278 | 0.220 | 0.250 |
|  | p-value |  | 0.142 | 0.003 | 0.671 | 0.216 | 0.114 | 0.235 | 0.014 | 0.053 | 0.027 |
|  | 95 % CI | Lower | -00.034 | 00.127 | -00.248 | -00.065 | -00.013 | -00.047 | -00.497 | 00.027 | 00.033 |
|  |  | Upper | 00.389 | 00.503 | 00.157 | 00.338 | 00.339 | 00.313 | 00.001 | 00.408 | 00.439 |
| **MEpurple** | Coefficient |  | -0.032 | 0.056 | -0.273 | -0.041 | -0.122 | -0.140 | -0.030 | 0.087 | -0.062 |
|  | p-value |  | 0.781 | 0.628 | 0.015 | 0.722 | 0.285 | 0.222 | 0.794 | 0.446 | 0.592 |
|  | 95 % CI | Lower | -00.21 | -00.164 | -00.447 | -00.239 | -00.335 | -00.338 | -00.226 | -00.145 | -00.282 |
|  |  | Upper | 00.165 | 00.267 | -00.081 | 00.159 | 00.072 | 00.052 | 00.186 | 00.29 | 00.153 |
| **MEblack** | Coefficient |  | -0.118 | -0.333 | -0.157 | -0.100 | -0.267 | -0.212 | 0.263 | -0.232 | -0.304 |
|  | p-value |  | 0.306 | 0.003 | 0.171 | 0.384 | 0.018 | 0.063 | 0.020 | 0.041 | 0.007 |
|  | 95 % CI | Lower | -00.335 | -00.512 | -00.327 | -00.318 | -00.501 | -00.431 | 00.015 | -00.441 | -00.477 |
|  |  | Upper | 00.097 | -00.133 | 00.054 | 00.099 | 00.003 | 00.016 | 00.487 | -00.014 | -00.129 |
| **MEdarkgrey** | Coefficient |  | -0.019 | -0.156 | -0.061 | -0.036 | -0.103 | -0.081 | 0.056 | -0.096 | -0.245 |
|  | p-value |  | 0.869 | 0.174 | 0.597 | 0.756 | 0.369 | 0.483 | 0.624 | 0.401 | 0.031 |
|  | 95 % CI | Lower | -00.246 | -00.395 | -00.266 | -00.284 | -00.355 | -00.336 | -00.131 | -00.322 | -00.435 |
|  |  | Upper | 00.19 | 00.066 | 00.154 | 00.175 | 00.12 | 00.143 | 00.245 | 00.102 | -00.048 |
| **MEgrey60** | Coefficient |  | -0.327 | -0.312 | -0.229 | -0.282 | -0.359 | -0.343 | 0.012 | -0.289 | -0.263 |
|  | p-value |  | 0.003 | 0.005 | 0.044 | 0.012 | 0.001 | 0.002 | 0.914 | 0.010 | 0.020 |
|  | 95 % CI | Lower | -00.55 | -00.499 | -00.411 | -00.506 | -00.54 | -00.515 | -00.163 | -00.522 | -00.478 |
|  |  | Upper | -00.109 | -00.107 | -00.006 | -00.055 | -00.154 | -00.144 | 00.196 | -00.048 | -00.044 |
| **MEcyan** | Coefficient |  | -0.291 | -0.374 | -0.164 | -0.273 | -0.366 | -0.329 | 0.214 | -0.192 | -0.243 |
|  | p-value |  | 0.010 | 0.001 | 0.150 | 0.016 | 0.001 | 0.003 | 0.061 | 0.093 | 0.032 |
|  | 95 % CI | Lower | -00.451 | -00.54 | -00.334 | -00.478 | -00.536 | -00.512 | 00.014 | -00.377 | -00.433 |
|  |  | Upper | -00.121 | -00.205 | 00.033 | -00.047 | -00.164 | -00.124 | 00.415 | -00.031 | -00.031 |
| **MEsalmon** | Coefficient |  | -0.181 | -0.092 | -0.046 | -0.138 | -0.129 | -0.129 | -0.061 | -0.071 | 0.017 |
|  | p-value |  | 0.113 | 0.425 | 0.692 | 0.228 | 0.260 | 0.262 | 0.597 | 0.538 | 0.882 |
|  | 95 % CI | Lower | -00.356 | -00.305 | -00.23 | -00.349 | -00.336 | -00.326 | -00.329 | -00.263 | -00.198 |
|  |  | Upper | 00.007 | 00.108 | 00.129 | 00.099 | 00.104 | 00.1 | 00.24 | 00.109 | 00.224 |
| **MEblue** | Coefficient |  | -0.283 | -0.207 | -0.287 | -0.294 | -0.332 | -0.346 | -0.011 | -0.061 | -0.179 |
|  | p-value |  | 0.012 | 0.070 | 0.011 | 0.009 | 0.003 | 0.002 | 0.922 | 0.594 | 0.117 |
|  | 95 % CI | Lower | -00.447 | -00.42 | -00.448 | -00.493 | -00.508 | -00.52 | -00.339 | -00.274 | -00.388 |
|  |  | Upper | -00.128 | -00.014 | -00.096 | -00.08 | -00.131 | -00.145 | 00.339 | 00.118 | 00.014 |
| **MEgreen** | Coefficient |  | -0.320 | -0.411 | -0.389 | -0.306 | -0.482 | -0.451 | 0.198 | -0.200 | -0.320 |
|  | p-value |  | 0.004 | 0.000 | 0.000 | 0.006 | 0.000 | 0.000 | 0.083 | 0.078 | 0.004 |
|  | 95 % CI | Lower | -00.473 | -00.581 | -00.563 | -00.5 | -00.659 | -00.628 | -00.019 | -00.421 | -00.517 |
|  |  | Upper | -00.15 | -00.24 | -00.184 | -00.112 | -00.257 | -00.262 | 00.42 | 00.003 | -00.132 |
| **MEdarkgreen** | Coefficient |  | 0.227 | 0.311 | 0.064 | 0.203 | 0.256 | 0.223 | -0.279 | 0.327 | 0.385 |
|  | p-value |  | 0.045 | 0.006 | 0.576 | 0.075 | 0.023 | 0.049 | 0.013 | 0.003 | 0.000 |
|  | 95 % CI | Lower | 00.039 | 00.137 | -00.161 | 00.016 | 00.058 | 00.034 | -00.459 | 00.092 | 00.195 |
|  |  | Upper | 00.397 | 00.473 | 00.264 | 00.412 | 00.431 | 00.392 | -00.109 | 00.527 | 00.546 |
| **MElightyellow** | Coefficient |  | 0.231 | 0.126 | 0.042 | 0.180 | 0.234 | 0.199 | -0.107 | 0.097 | 0.250 |
|  | p-value |  | 0.042 | 0.272 | 0.715 | 0.114 | 0.039 | 0.081 | 0.350 | 0.396 | 0.027 |
|  | 95 % CI | Lower | 00.001 | -00.063 | -00.155 | -00.04 | 00.03 | -00.016 | -00.331 | -00.137 | 00.058 |
|  |  | Upper | 00.454 | 00.329 | 00.246 | 00.391 | 00.421 | 00.401 | 00.11 | 00.345 | 00.429 |
| **MEred** | Coefficient |  | 0.399 | 0.370 | 0.232 | 0.380 | 0.387 | 0.394 | -0.186 | 0.313 | 0.469 |
|  | p-value |  | 0.000 | 0.001 | 0.041 | 0.001 | 0.000 | 0.000 | 0.103 | 0.005 | 0.000 |
|  | 95 % CI | Lower | 00.218 | 00.189 | 00.027 | 00.187 | 00.208 | 00.209 | -00.401 | 00.094 | 00.322 |
|  |  | Upper | 00.555 | 00.528 | 00.424 | 00.564 | 00.537 | 00.565 | 00.049 | 00.519 | 00.59 |
| **MEdarkturquoise** | Coefficient |  | 0.064 | -0.036 | 0.206 | -0.082 | 0.012 | 0.022 | 0.173 | -0.123 | -0.053 |
|  | p-value |  | 0.578 | 0.757 | 0.070 | 0.477 | 0.918 | 0.851 | 0.131 | 0.285 | 0.644 |
|  | 95 % CI | Lower | -00.132 | -00.269 | 00.005 | -00.296 | -00.224 | -00.195 | -00.012 | -00.306 | -00.259 |
|  |  | Upper | 00.256 | 00.195 | 00.405 | 00.171 | 00.259 | 00.27 | 00.361 | 00.074 | 00.165 |
| **MEroyalblue** | Coefficient |  | 0.023 | -0.138 | 0.101 | 0.055 | -0.030 | 0.027 | 0.137 | -0.127 | -0.109 |
|  | p-value |  | 0.842 | 0.228 | 0.380 | 0.634 | 0.793 | 0.818 | 0.233 | 0.266 | 0.342 |
|  | 95 % CI | Lower | -00.222 | -00.355 | -00.091 | -00.179 | -00.262 | -00.209 | -00.097 | -00.352 | -00.305 |
|  |  | Upper | 00.257 | 00.077 | 00.259 | 00.281 | 00.169 | 00.225 | 00.302 | 00.078 | 00.087 |
| **MEtan** | Coefficient |  | -0.010 | -0.032 | 0.147 | 0.027 | 0.031 | 0.059 | -0.087 | -0.128 | 0.033 |
|  | p-value |  | 0.934 | 0.782 | 0.200 | 0.812 | 0.789 | 0.610 | 0.448 | 0.264 | 0.774 |
|  | 95 % CI | Lower | -00.252 | -00.238 | -00.05 | -00.198 | -00.182 | -00.157 | -00.263 | -00.354 | -00.172 |
|  |  | Upper | 00.229 | 00.168 | 00.325 | 00.255 | 00.247 | 00.27 | 00.096 | 00.104 | 00.232 |
| **MEmidnightblue** | Coefficient |  | 0.164 | 0.087 | 0.155 | 0.191 | 0.149 | 0.183 | 0.035 | -0.023 | 0.137 |
|  | p-value |  | 0.152 | 0.451 | 0.177 | 0.094 | 0.193 | 0.109 | 0.758 | 0.842 | 0.230 |
|  | 95 % CI | Lower | -00.076 | -00.12 | -00.04 | -00.025 | -00.06 | -00.02 | -00.192 | -00.242 | -00.045 |
|  |  | Upper | 00.386 | 00.294 | 00.324 | 00.417 | 00.339 | 00.388 | 00.26 | 00.233 | 00.323 |
| **MEpink** | Coefficient |  | -0.140 | -0.151 | -0.056 | -0.085 | -0.164 | -0.130 | 0.170 | -0.130 | -0.012 |
|  | p-value |  | 0.221 | 0.188 | 0.625 | 0.459 | 0.152 | 0.258 | 0.137 | 0.256 | 0.914 |
|  | 95 % CI | Lower | -00.35 | -00.346 | -00.252 | -00.317 | -00.36 | -00.325 | 00.024 | -00.331 | -00.233 |
|  |  | Upper | 00.089 | 00.051 | 00.13 | 00.192 | 00.051 | 00.118 | 00.328 | 00.08 | 00.214 |
| **MEyellow** | Coefficient |  | 0.055 | 0.180 | 0.078 | 0.105 | 0.145 | 0.132 | -0.148 | 0.045 | 0.261 |
|  | p-value |  | 0.634 | 0.115 | 0.499 | 0.361 | 0.206 | 0.250 | 0.196 | 0.694 | 0.021 |
|  | 95 % CI | Lower | -00.163 | -00.034 | -00.134 | -00.117 | -00.078 | -00.082 | -00.331 | -00.169 | 00.057 |
|  |  | Upper | 00.273 | 00.372 | 00.276 | 00.364 | 00.36 | 00.355 | 00.084 | 00.29 | 00.432 |

**Supplementary Table 3**. Description of the modules significantly associated with clinical data. Table shows for each module, the number of genes included, the number of hub genes and the number of genes overlapping to other studies.

|  | **Module** | **Genes^1^** | **Hub Genes^2^** | **Overlap Genes^3^** | **Genes^4^** |
| --- | --- | --- | --- | --- | --- |
| Cluster 1 | Blue | 4329 | 1058 | 282 | VEZT, SCOC, TRAF3IP3, XYLT2, GPATCH2, PREP, CBLB, ICAM5, IKZF5, HERC4, ISLR, CNST, VSIG2, PPAT, GPBP1, CHORDC1, BBX, CPNE3, FBXO8, ACAD11, SCAPER, VPS36, PRKACB, TMPO, SEC63, MCCC2, ZNF283, COG5, CMC2, TYW3, SACM1L, IFT80, GTPBP8, MED7, MRPS18C, RNF125, MTHFD1, KCNQ1, RUFY2, FAR1, FAM76B, UPRT, CLOCK, MTMR1, TMEM87A, MTMR2, SMIM5, STXBP3, ABCB7, TXK, CWF19L2, VPS26A, ARPP19, RPAP2, TTBK2, MTDH, FXR1, SNX4, HSPH1, PDCD10, VPS8, MIER1, N4BP2, SEC31A, NUS1, EGR4, TIPARP, SETDB2, THAP12, NBEAL1, LARP7, BBIP1, LARP4, GLUD1, PPP1R1A, NDUFAF6, LPAR6, FUBP1, WDR5B, TRMT13, SFXN1, CYSTM1, ATM, CDC42SE2, PHF3, CEP57, HBS1L, SMG1, FASTKD1, PHF20, PYROXD1, PHF6, EPM2AIP1, ZNF808, TRIM4, OTUD6B, TMED2, EMB, BBS7, ENOPH1, RAB8B, RALGPS2, UPF2, ZNF483, SHPRH, CNOT6L, COMMD2, SLC39A10, ZDHHC17, SIRT1, WDR33, CTU1, CLDN15, AEBP2, HNRNPH1, BANK1, MED21, PIGK, POT1, CD47, MICU2, RAPGEF6, RAPGEF6, ERGIC2, EXOC1, FYTTD1, DYNC1I2, TMF1, ZDHHC20, STAU2, USO1, MRPS30, ADAM28, NRAS, ZFP14, MGAT5, STAT4, MFN1, CCT8, BRD7, METTL8, RAE1, SF3B1, TSNAX, MAP3K1, SLF2, YIPF5, IGIP, ATAD2, WDR19, RPA1, ORMDL1, GTF2H1, PUS7L, MYO9A, KTN1, ZNF33B, ZNF33A, SPCS3, TAOK3, KLHL5, XPOT, PRKRA, SNX14, TTC3, CAPZA2, CCDC7, CCDC7, RLIM, GOPC, LAMTOR1, CD69, ZNF354B, OTUD4, POGLUT1, ZNF570, FAM13B, ATP8A1, TMEM167A, FMR1, ZBTB26, GIMAP2, LTN1, ZFAND1, HNMT, GIMAP7, FBXO22, BZW1, DOCK10, DOCK11, CHCHD7, CWC22, PAPOLA, SMARCAD1, KPNA5, C18orf25, SLC25A46, ZNF441, ANKRD36C, SLC30A6, USP8, ZNF680, ZGRF1, SPICE1, ZNF98, CDYL, CASD1, PIAS2, SHQ1, ZNF92, MAP7D3, HAT1, SREK1, SRSF6, IDI1, TXNDC9, INSIG2, COPB1, UBR3, GPATCH2L, LIN7C, RLF, DPP4, ATXN3, ODF2L, TMEM245, MAN2A1, TTC21B, G3BP1, SLC38A9, AP4S1, SLC38A2, SOCS5, ZNF782, DR1, ACBD5, TIA1, VTA1, GFPT1, ZBTB10, HELZ, DNAJC13, FOXN2, CLK4, CNOT4, DIAPH2, DLG1, APPBP2, P4HA1, JMY, UBE2N, UBA3, TPP2, CYCS, ZNF654, TMEM19, GALK1, RBM27, RBM27, KDM5A, ZNF770, DDX46, USP33, DOCK7, PIK3C2A, HS2ST1, NADK2, METTL14, PTER, RB1CC1, SCRN3, MFSD1, POLK, TOPBP1, DNM1L, TTC37, HSP90AA1, DIS3, VPS13C, DCXR, LANCL1, ARAP2, SLC25A16, RABEP1, TRAF3, RASA1, TRAF5, ATG4C, TRIM59, ZNF512, ZMYND11, FAN1, BIRC3, SLC25A13, ATG4D, SLFN5, USP16, RRN3, RGS14, ROCK2, RPE, RNF6, PPM1K, CLCN3, NR2C1, NT5DC1, IFT74, CAND1, TNKS2, VPS50, ZNF507, OXCT1, ZNF626, RAB29, FEM1C, HNRNPA1, ATP9B, S100PBP, EIF4B, TRIM44, CLASP2, TTC14, SLC12A2, SMOX, MGA, CYP51A1, DDHD2, CDC5L, NAP1L1, LYSMD3, MGAT4A, ZNF736, VPS41, ERCC8, ERCC5, FAM98B, SNRNP48, NAA15, FAM98A, PPIL4, TRMT61A, PTPN2 |
|  | Green | 536 | 261 | 72 |  |
|  | Cyan | 151 | 12 | 1 |  |
| Cluster 2 | Red | 402 | 71 | 25 | ZER1, RBM38, MAP2K3, FOXO3, XK, SLC14A1, SLC38A5, NFE2, TAL1, MAP2K7, PLEK2, UBL7, FAXDC2, ALAS2, SLC48A1, BLVRB, NINJ2, ZDHHC18, LRP10, SBNO2, NFAM1, MMP25, FPR1, SLC11A1, APOBEC3A, TNFRSF1A, VSIR, ERGIC1, LRRC4, RGL2, PILRA, GNB2, TMEM120A, AGRN, PIP5K1A, COL16A1, TMEM54, SYT2, PROC, FNDC4, LAMB2, FGFR4, MAPK9, BCAP29, TRAPPC9, SAPCD2, NUDT10, RPP30, COMTD1, AVPI1, ROM1, SNX32, TRAF6, KCNA5, VWF, ANAPC7, COL4A2, SLC46A3, MYH7, SNW1, GREM1, TNFRSF12A, ENKD1, SERPINF2, PPP1R1B, RASD1, MPP3, C1QL1, FDXR, CERS4, CBARP, SPC24, PPP1R14A, PLEKHA4, C19orf25, COL20A1, SLC52A3, NECAB3, GHRH, RIPOR3, POFUT2, FKBP8, SLC38A5, JAZF1, UBL7, SLC48A1, RILP, MAP2K2, COL16A1, FNDC4, EGFL7, SAPCD2, KREMEN2, PPP1R1B, SHD, TTYH1, NECAB3, CLCN3, FOXN2, FXR1, PIK3C2A, SF3B1, VSIG2, ZNF512, TTC14 |
|  | Turquoise | 5637 | 281 | 62 |  |
|  | Magenta | 314 | 51 | 15 |  |

^1^The number of genes included in the module during the WGCNA process

^2^Hub genes in the module according to the gene significance (GS>0.3) and their module membership (MM>0.8)

^3^Hub genes of the module that showed overlap with the external validation datasets

^4^Gene symbols of Hub genes of the Cluster that showed overlap

**Supplementary Figure 1**. WGCNA network and module detection. **(A)** (Sample clustering was conducted to detect outliers. Red color identify the excluded sample **(B)** Selection of the soft-thresholding powers. The left panel showed the scale-free fit index versus soft-thresholding power. The right panel displayed the mean connectivity versus soft-thresholding power. Power 6 was chose, for which the fit index curve flattens out upon reaching a high value (>0.8) **(C)** Clustering dendrogram of genes based on a dissimilarity measure (1-TOM), which was then used to group genes into 25 modules in baseline samples. The branches correspond to modules of highly interconnected groups of genes. The height (y-axis) indicates the co-expression distance and the x-axis corresponds to genes. Colors represent the 25 different modules along with gray indicating genes that could not be assigned to any module.


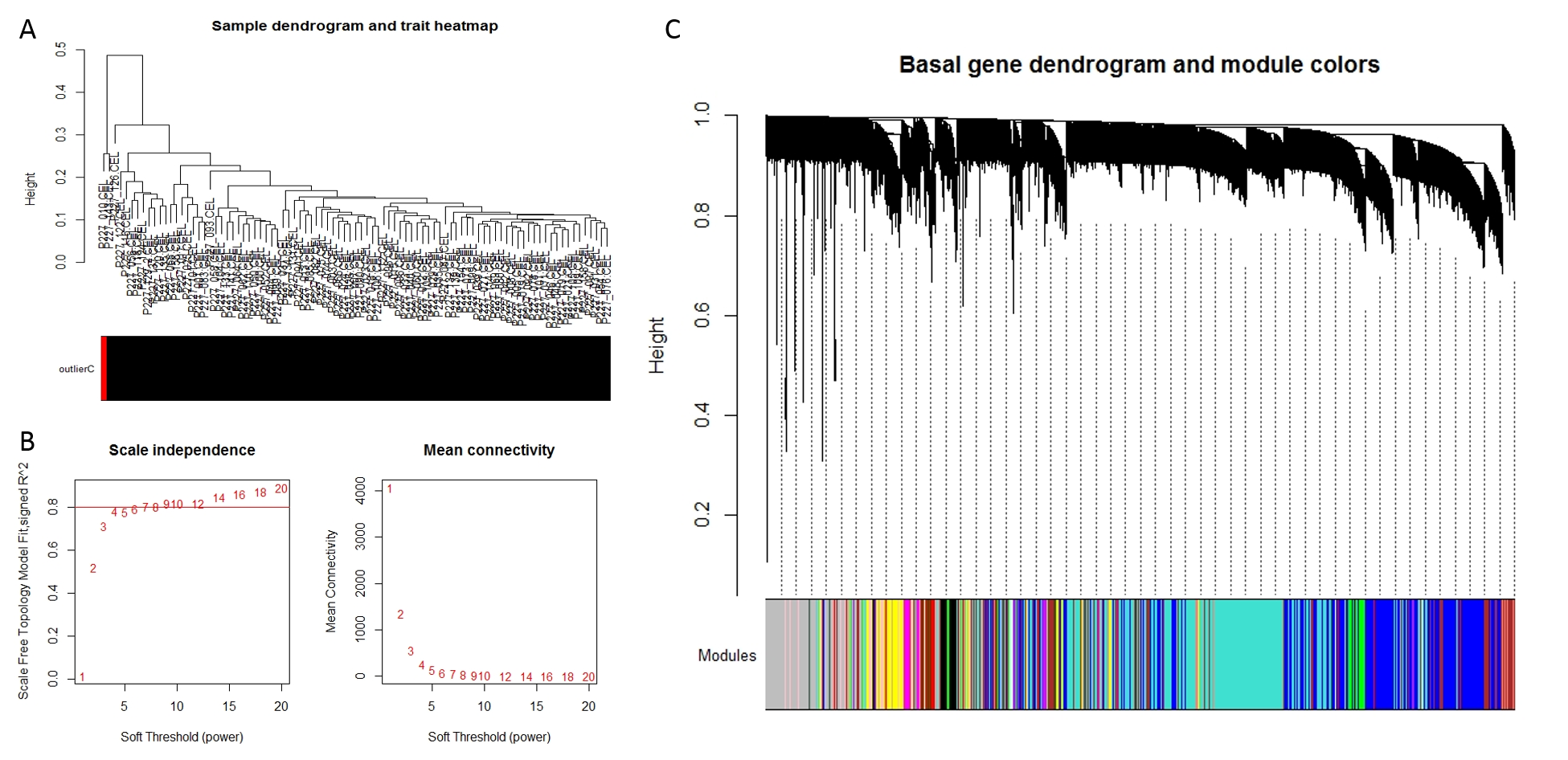


**Supplementary Figure 2.** Module stability study using resampling of microarray samples. The upper panel shows the hierarchical clustering dendrogram of all probe sets. Branches of the dendrogram correspond to modules, identified by solid blocks of colors in the color row. Color rows beneath the first row indicate module assignments obtained from networks based on resampled sets of microarray samples.


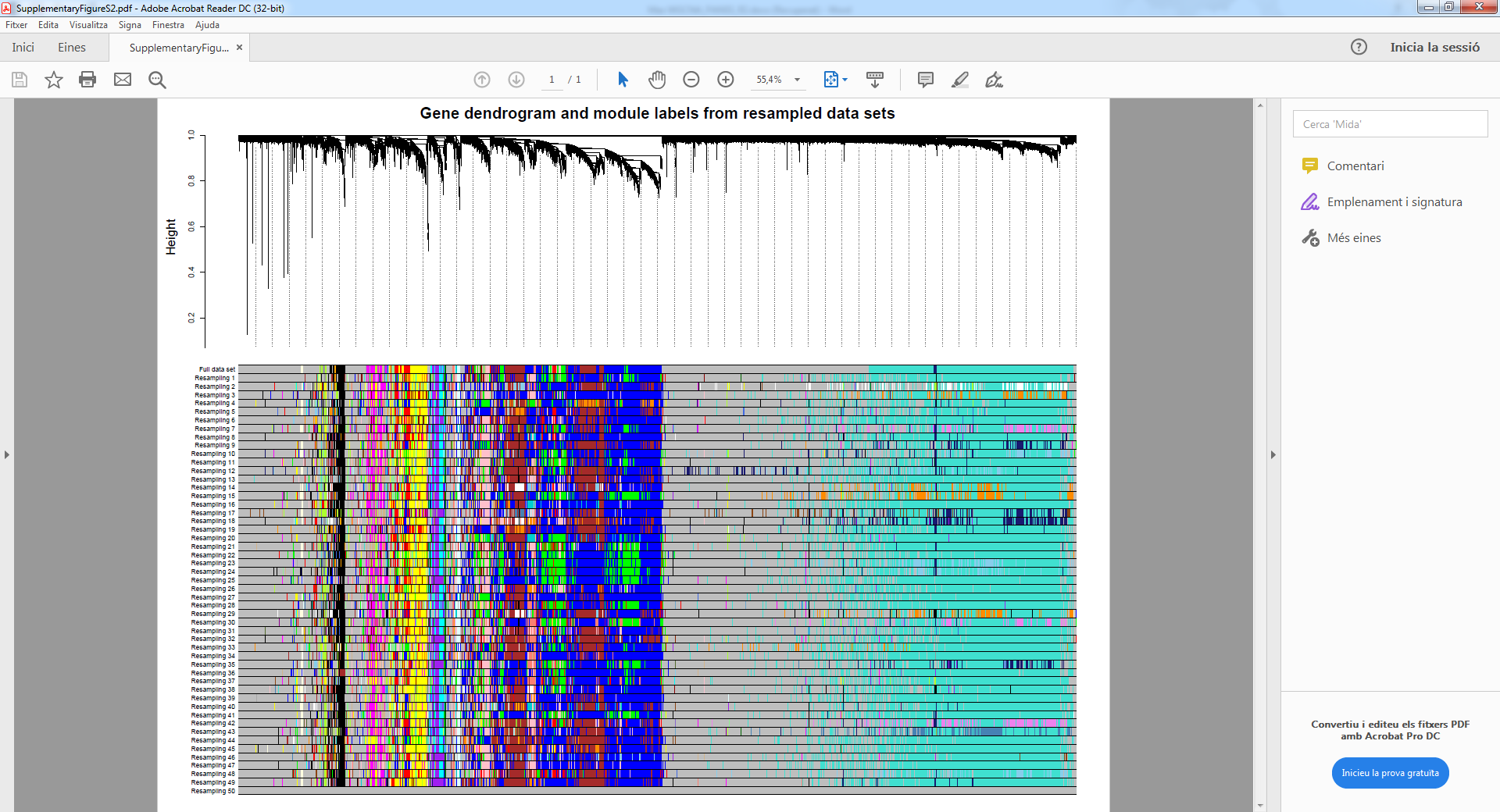

Supplement: Supplementary file 1 — Supplementary Information [file 41537_2022_215_MOESM1_ESM.docx]
